# Extent of Food Processing and Risk of Prostate Cancer: The PROtEuS Study in Montreal, Canada

**DOI:** 10.3390/nu12030637

**Published:** 2020-02-28

**Authors:** Karine Trudeau, Marie-Claude Rousseau, Marie-Élise Parent

**Affiliations:** 1 Epidemiology and Biostatistics Unit, Centre Armand-Frappier Santé Biotechnologie, Institut national de la recherche scientifique, University of Quebec, Laval, QC H7V 1B7, Canada; karine.trudeau@iaf.inrs.ca (K.T.); marie-claude.rousseau@iaf.inrs.ca (M.-C.R.); 2 School of Public Health, Department of Social and Preventive Medicine, University of Montreal, Montreal, QC H3N 1X9, Canada; 3 University of Montreal Hospital Research Centre, Montreal, QC H2X 0A9, Canada

**Keywords:** prostate cancer, case-control study, NOVA, food processing, ultra-processed foods, unprocessed or minimally processed foods, processed culinary ingredients, processed foods

## Abstract

We studied the association between food intake, based on the extent of processing, and prostate cancer risk in a population-based case-control study conducted in Montreal, Canada in 2005–2012. Incident prostate cancer cases (*n* = 1919) aged ≤75 years were histologically confirmed. Population controls (*n* = 1991) were randomly selected from the electoral list and frequency-matched to cases by age (±5 years). A 63-item food frequency questionnaire focusing on the two years prior to diagnosis/interview was administered by interviewers. The NOVA classification was used to categorize foods based on processing level. Unconditional logistic regression estimated the association between food intake and prostate cancer risk, adjusting for age, education, ethnicity, family history, and timing of last prostate cancer screening. Consumption of unprocessed or minimally processed foods showed a slight, inverse association (Odd ratio [OR] 0.86, 95% confidence interval [CI] 0.70–1.07; highest vs. lowest quartile) with prostate cancer. An increased risk was observed with higher intake of processed foods (OR 1.29, 95%CI 1.05–1.59; highest vs. lowest quartile), but not with consumption of ultra-processed food and drinks. The associations with unprocessed/minimally processed foods and processed foods were slightly more pronounced for high-grade cancers (ORs 0.80 and 1.33, respectively). Findings suggest that food processing may influence prostate cancer risk.

## 1. Introduction

Prostate cancer was the most commonly diagnosed cancer, after non-melanoma skin cancer, among Canadian men in 2019, accounting for 22,900 of estimated new cases [1]. It was the third cause of cancer death with 4100 estimated deaths. The burden will increase in this country, with 33,063 new cases projected for 2023 [2]. The only definitively established risk factors for prostate cancer are advancing age, family history of prostate cancer, and African ancestry [3,4,5]. Despite extensive research to understand its etiology, there are still no known modifiable risk factors for this cancer [5]. The geographic distribution of the incidence of prostate cancer and studies of migrant populations strongly suggest that its etiology is at least partially influenced by environmental factors and lifestyle habits, possibly diet [6]. Most previous studies focusing on the latter have evaluated the role of a single food or nutrient at a time, with contradictory findings [7,8]. According to the World Cancer Research Fund, there is only limited evidence that dairy products, diets high in calcium, low-plasma tocopherol concentrations, and low plasma selenium concentrations increase the risk of prostate cancer [5].

Several factors can influence nutritional quality, including education and occupation. Although education may help make healthier choices, busy lifestyles can push people with lower or higher educational levels to consume ready-to-eat meals, including ultra-transformed food [9]. Ultra-transformed foods are formulations made mostly or entirely from substances derived from foods and additives, with little if any intact unprocessed or minimally processed food [10,11]. Their consumption might be linked to chronic conditions such as type 2 diabetes, obesity and cancers [12]. Surveys in Europe, the US, Canada, Brazil and New Zealand on individual food intake, household food expenses, or supermarket sales show that ultra-processed food products represent 25% to 50% of the total daily energy intake [13,14]. Understanding the health impact of ultra-processed foods has gained in interest as the global consumption of ultra-processed foods increases [15]. To our knowledge, only one published study to date has reported on the frequency of ultra-processed foods consumption in relation with cancer risk. It found no association with prostate cancer, albeit based on relatively few cases [13]. A recent study suggests a higher risk of all-cause mortality associated with a greater consumption of ultra-processed foods [16].

Several aspects of foods undergoing processing justify studying them in relation to cancer risk: their nutritional composition (e.g., high contents of saturated fat, sugar, salt), the presence of some contaminants and additives (e.g., acrylamide, heterocyclic amines, polycyclic aromatic hydrocarbons, emulsifiers, artificial sweeteners), and their packaging (e.g., bisphenol A) [17]. By contrast, the consumption of unprocessed foods such as fruits and vegetables has been linked with a reduced risk of aerodigestive cancers [18].

The objective of the present study was to evaluate the association between the extent of processing of food in the diet and prostate cancer risk, both overall and by tumour aggressiveness. 

## 2. Materials and Methods 

### 2.1. Study Population

We used data from the Prostate Cancer and Environment Study (PROtEuS), a large population-based case-control study conducted in Montreal, Canada aimed at identifying environmental, occupational, lifestyle and genetic factors involved in the development of prostate cancer. This study has been described previously [19]. In brief, eligible cases and controls were Canadian citizens registered on the provincial electoral list, residents of the Montreal metropolitan area and aged <76 years at diagnosis or interview (index date). Cases were diagnosed with primary, histologically confirmed prostate cancer between September 2005 and December 2009. They were actively ascertained through pathology departments across 7 of the 9 French-language hospitals that diagnose prostate cancer in the Montreal area, thus covering at least 80% of all cases diagnosed in the area according to registry information. Concomitantly, population controls were randomly selected from the electoral list among French-speaking men residing in the same geographical area as cases, and were frequency-matched to cases by age (±5 years). Eligible controls had no history of prostate cancer. In all, 1932 cases and 2036 controls aged 39–75 years were recruited. Participation rates were 79% among eligible cases and 56% among eligible controls. Using 2006 Census data, we established that the percentages of subjects living in areas with a greater proportion of recent immigrants were 5% and 6%, for participants and non-participants, respectively. Corresponding values respectively were 7% and 7% for a higher unemployment rate, 19% and 20% of adults without a high school diploma, and 22% and 25% in the lowest quintile of household income. These observations are in line with a very slight trend towards higher socio-economic status among participants, a feature commonly encountered, but reassure against the possibility of a strong selection bias. Proxy respondents, usually the spouse, provided information for 3% of cases and 4% of controls.

PROtEuS was approved by the Comité d’éthique en recherche avec les êtres humains of the Institut national de la recherche scientifique (CÉR-02-036, from 8 October 2002 until the present day), as well as by the ethics boards of all participating hospitals. All subjects provided written informed consent.

### 2.2. Data Collection

Face-to-face interviews were conducted between 2005 and 2012, mainly in the house of participants, collecting a wide range of information on sociodemographic, environmental, lifestyle, and medical factors. Of particular interest here, these included education, ethnicity, family history of prostate cancer, diet, alcohol, coffee and tea consumption, and smoking history. In addition, the prostate cancer screening history by prostatic specific antigen (PSA) and/or digital rectal exam (DRE) was collected. Tumour Gleason scores were extracted from pathology reports of prostate biopsies at diagnosis.

Dietary information was collected using a 63-item food frequency questionnaire (FFQ) based on a validated instrument used by the Canadian Cancer Registries Epidemiology Research Group, with slight modifications to reflect the specificity of the study population [20]. Cases and controls were asked to report their consumption of food at home, at work, and at restaurants two years prior to the index date. The consumption of food items, expressed in commonly-used portions, was recorded in terms of the frequency of use per day, week or month. Dietary data were missing for 13 cases and 3 controls, and 42 controls became cases of prostate cancer during the ascertainment period, leaving 1919 cases and 1991 controls for the analysis. Participants were asked how many months per year they ate various fresh fruits in order to take into account seasonal variations. Data on lifelong use of coffee, black tea, green tea, beer, wine, and spirits were collected; levels of use two years before the index date were retained for the present analyses.

### 2.3. Statistical Analysis

Each item from the FFQ and the beverage questions (coffee, black tea, green tea, beer, wine and spirits consumption) was classified into one of four groups based on the NOVA food classification [10,21], as shown in Table 1.

The first group contains unprocessed or minimally processed foods such as fresh fruits and vegetables, pasta, and pasteurized milk. The second group includes processed culinary ingredients such as salt, sugar, maple syrup, vegetable oils, and butter. The third group consists of processed foods such as cheese, fruits in syrup, beer, cider, and wine. The fourth group is constituted of ultra-processed foods and drink products such as breakfast cereals, soft drinks, fruit yogurt, ice cream, chocolate, candies, pasta dishes, and pizza. As we only had one item in group 2 (butter), we excluded this grouping from our analysis.

We calculated a consumption score for each of groups 1, 3, and 4. This was done by summing the weekly frequency of use of foods in the FFQ corresponding to a given group, then dividing by the frequency of use of all FFQ food items consumed per week. Scores were categorized into quartiles based on distributions among controls.

Unconditional logistic regression was used to estimate odds ratios (ORs) and 95% confidence intervals (CIs) for the association between each food group score, in quartiles and prostate cancer risk, overall and by tumour aggressiveness. The latter was defined using the Gleason score. Risk of low-grade (Gleason scores <7 or 7 with a primary grade of 3) and high-grade (Gleason scores of 7 with a primary grade of 4, or higher) cancers [22,23] were estimated using polytomous models.

**Figure 1:**
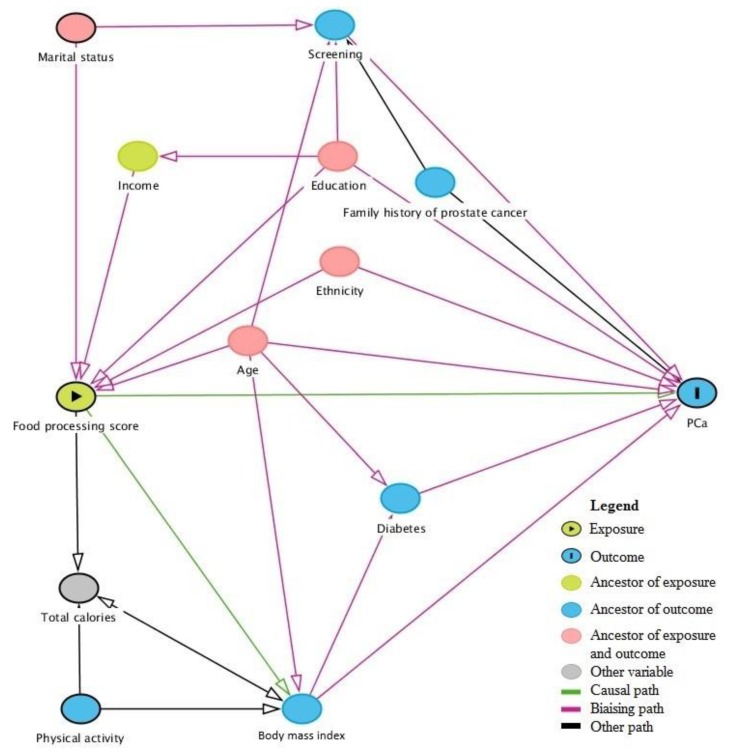
Directed acyclic graph (DAG) for the association between food processing and prostate cancer.

Figure 1 presents a directed acyclic graph (DAG) for the association between the extent of food processing in the diet and prostate cancer. Variables considered in the DAG include age at diagnosis for cases or age at interview for controls (continuous), ethnicity (Asian, Sub-Saharan, European, Greater Middle Eastern, Latino), education (elementary or less, high school, college, university, other), first-degree family history of prostate cancer (yes, no, do not know), timing of the last prostate screening by PSA and/or DRE (≤2 years before index date, >2 years before index date, never screened, do not know), marital status (married and common law, separated and divorced, single, widower, member of a religious order, do not know), income (<20,000$CAD, 20,000–29,999$CAD, 30,000–49,999$CAD, 50,000–79,999$CAD, >80,000$CAD), diabetes (yes, no, do not know), body mass index (BMI, continuous), total calories (kcal/day, continuous), and physical activity (not very active, moderately active, and very active). The total effect (shown in pink in Figure 1) closes all biasing paths and leaves all causal paths opened. A minimal model included age, education, ethnicity, and marital status. We chose to present here results based on a more etiologically relevant model, which includes age, education, ethnicity, first-degree family history of prostate cancer, and timing of last prostate screening test. Continuous variables were treated as such after confirming the linearity of the logits, or as categorical otherwise. The covariates included in the adjusted model presented few missing values, which were modeled as a separate category.

In order to evaluate the possible impact of latent prostate cancers in our control series, we conducted a sensitivity analysis, excluding controls that were not screened for prostate cancer within the previous two years.

## 3. Results

### 3.1. Study Population 

The characteristics of 1919 cases and 1991 controls are displayed in Table 2.

Compared to controls, cases were slightly younger by about one year (owing to the longer time to recruit controls), less educated, more often of European or African ancestries, while less often of Asian or Greater Middle Eastern ancestries, and a greater proportion had a first-degree family history of prostate cancer. Most controls (76%) had gone through a screening test carried out by PSA and/or DRE within the previous two years. There were little differences in terms of family income, education, cigarette smoking, physical activity, and total calories intake two years before the index date.

### 3.2. Association between Food Classification and Prostate Cancer Risk

Table 3 present results for the association between the consumption of foods according to different categories of food processing and the risk of prostate cancer, overall and by disease aggressiveness, after adjusting for potential confounders identified in the DAG. 

Consumption of unprocessed or minimally processed foods showed a slight, inverse association with prostate cancer overall (OR = 0.86, 95%CI 0.70–1.07, comparing the fourth quartile of the consumption score to the first). By contrast, a higher consumption of processed foods was associated with a higher risk of overall prostate cancer (OR = 1.29 and 95%CI = 1.05–1.59, after comparing the fourth to the first quartile). Finally, consumption of ultra-processed foods and drinks was not associated with risk.

Results from analyses based on cancer aggressiveness showed a slightly lower risk of high-grade cancers with consumption of unprocessed/minimally processed foods, as compared to low-grade tumours. Contrastingly, the risk estimate associated with higher consumption of processed foods was slightly more pronounced for high- than for low-grade cancers.

In the sensitivity analysis, we excluded controls not screened for prostate cancer in the previous two years (24%), which were more likely to have an undiagnosed prostate cancer than those who had recently been screened, meaning results remained largely unchanged (data not shown).

## 4. Discussion

In this study, we observed that consumption of processed foods was associated with a higher risk of prostate cancer. Higher levels of consumption of unprocessed or minimally processed foods was associated with a slightly lower risk of this cancer. To our knowledge, this is the first study to document such associations.

As the consumption of ultra-processed food increases in industrialized countries, such as Canada [14], the overall quality of diets decreases. Based on this, we would have expected that the consumption of ultra-processed foods would have been associated with an increased risk of prostate cancer. However, similarly to our study, the NutriNet-Santé prospective cohort of 104,980 participants, including 281 incident cases of prostate cancer, observed no association [13]. In the latter study, the only one to assess links with cancer, ultra-processed food consumption was associated with higher overall and breast cancer risks. We expanded on this by examining not only the ultra-processed NOVA food group but also the unprocessed/minimally processed and processed food groups.

We found that consumption of processed foods was associated with a higher risk of prostate cancer. This is an original finding. Processed foods are industrial products made by adding salt, sugar, fat and other foods found in group 2 (processed culinary ingredients) to unprocessed or minimally processed foods (group 1). Thus, our result pertaining to processed foods might be explained by the presence of fat, salt, sugar, in processed food along with low fibre and vitamin density. The absence of an association with ultra-processed foods in our study may possibly indicate that the food items listed in our FFQ under this food group contained limited amounts of contaminants and additives such as acrylamide, heterocyclic amines, polycyclic aromatic hydrocarbons, emulsifiers, artificial sweeteners, and bisphenol A, although it is not possible for us to confirm this.

### 4.1. Potential Mechanisms

It is noteworthy that consumption of unprocessed or minimally processed foods in our population included a lot of fruits and vegetables, meat, poultry, fish or seafood, rice, and milk. This would result in a diet rich in vitamins and antioxidants, with better nutritional value than a diet rich in ultra-processed food and drink products [14]. Vitamins are essential nutrients for human metabolism [24]. In in vitro and in vivo experiments [25], vitamin A and retinoids were shown to modulate malignant cell growth by apoptosis, growth arrest and redifferentiation and in prostate cancer, the homeostasis of vitamin A and retinoids is altered [26]. Vitamin C is an antioxidant that prevents the formation of reactive oxygen species (ROS), and has an effect on tumor cells [27,28]. It is possible that 1,25-dihydroxy vitamin D3 decreases cell proliferation in the prostate [5]. Vitamin E might play a role in cancer defenses by preventing DNA damage, enhancing DNA repair, and by enhancing the immune response [5]. Vitamin K may initiate apoptosis through a nonoxidative mechanism and might involve transcription factors [24,29]. Finally, polyphenols, which include a wide family of molecules bearing multiple phenolic rings, such as flavonoids and phenolic acids, may have a role in the prevention of cancer by reducing oxidative stress and inflammation [30,31].

On the other hand, a higher consumption of processed foods was associated with a higher risk of overall prostate cancer. According to the World Cancer Research Fund and the American Institute for Research on Cancer, there is currently limited, inconclusive evidence that saturated fatty acids, mono-unsaturated fatty acids, and poly-unsaturated fatty acids influence the risk of prostate cancer [5]. Nevertheless, prostate cancer development could be related to abnormal steroid receptor signaling which are composed of lipids. Indeed, recent studies using human prostate regeneration models have shown a carcinogenic effect of steroids [32]. Thus, a diet rich in fat, compatible with a diet high in processed foods, could perhaps contribute to the pathogenesis of prostate cancer [33].

### 4.2. Methodological Considerations

The sample size of our study, including 1919 incident cases, is a definite advantage, thereby improving the ability to detect potentially modest associations with dietary factors. Despite this, sub-group analyses such as those based on high-grade cancers can be hampered by limited statistical power. The participation rate was relatively good as compared to several previous similar investigations [34]. Nevertheless, non-responses could have introduced a selection bias, although comparisons of participants and non-participants in terms of census-derived indicators suggested that this would have been minimal.

Given the retrospective nature of the study, differential recall bias between cases and controls might have occurred, where participants could have been more attentive to their food consumption before their diagnosis, and over or under-reported it. The FFQ focused on the two years prior to the index date, thereby reflecting recent intakes. These would be especially relevant for cancer progression and for initiation if they reflected stable intakes over several years. While the FFQ necessarily entailed some degree of misclassification, it is a superior method to food records to assess usual food habits [35]. Misclassification might have occurred when categorizing items from the dietary questionnaire into the processed food group and the ultra-processed food and drink products group. Indeed, we decided to classify spaghetti, lasagna, or other pastas with tomato sauce into the processed food group because we assumed that these foods were a combination of unprocessed or minimally processed foods group and processed culinary ingredients group, but it might occur that these foods were ultra-processed in reality. If misclassification occurred, then the ORs for ultra-processed food would have leaned towards the null value. Another example is that we chose to classified yogurt into the ultra-processes food because we assumed that it was most often “fruit” yogurt with sugar instead of plain yogurt. As the plain yogurt is an unprocessed or minimally processed food, misclassification would have attenuated associations with ultra-processed foods.

From a nutritional point of view, an advantage of studying food processing through food groups is that it considers that foods are ingested together, as part of meals, rather than being consumed individually. Nutrients and food combinations can result in food synergy and interactions [36] which in turn may be associated with health and outcomes. Moreover, food processing may be more amenable to health promotion and disease prevention than individual nutrients, since food processing is more closely aligned with overall dietary habits. As a result, it may be more easily translated into public health recommendations.

## 5. Conclusions

Our study documents that higher intakes of processed foods are associated with an increased risk of prostate cancer. This is a noteworthy finding given that food processing is ubiquitous. There was also suggestion that consumption of unprocessed or minimally processed foods was associated with a lower risk of this cancer. Finally, these findings are important as they enhance the understanding of the relation between dietary habits and prostate cancer, which can inform public health recommendations.

## Figures and Tables

**Table 1:** NOVA food groups ^1^ and corresponding foods from the food frequency questionnaire in the Prostate Cancer and Environment Study (PROtEuS).

**NOVA Food Group 1: Unprocessed or Minimally Processed Foods**	
Examples of foods in NOVA group	Fresh fruits and vegetables, herbs, legumes, meat, poultry, fish, eggs, pasteurized milk, plain yogurt, tea, coffee, oats
Foods from the FFQ ^2^	Banana, apple, orange, peaches, apricots, cantaloupe, watermelon, berries, potatoes, sweet potatoes, legumes, broccoli, carrots, spinach, cabbage, cauliflower, dark lettuce, tomatoes, sweet red pepper, beef, pork, chicken, veal, lamb, liver, fish, eggs, rice, oatmeal, milk, cream, tea, coffee
**NOVA Food Group 2: Processed Culinary Ingredients**	
Examples of foods in NOVA group	Salt, sugar, maple syrup, vegetable oils, butter
Foods from the FFQ	Butter
**NOVA Food Group 3: Processed Foods**	
Examples of foods in NOVA group	Cheese, fruits in syrup, beer, cider, wine
Foods from the FFQ	Tomato soup, vegetable soup, tofu, meat grilled on the barbeque, spaghetti, cheese, nuts, beer, wine
**NOVA Food Group 4: Ultra-Processed Foods**	
Examples of foods in NOVA group	Breakfast cereals, soft drinks, spirits, fruit yogurt, ice cream, chocolate, candies, pasta dishes, pizza, sausages, pastries, cakes, pre-prepared pasta
Foods from the FFQ	Fried potatoes, ketchup, salsa, salad dressing, mayonnaise, hot-dog, cheese macaroni, pizza, cookies, muffins, white bread, brown bread, breakfast cereal, real juice, tomato juice, dark carbonated soft, drinks, other carbonated soft drinks, margarine, fried food, chips, chocolate, yogurt, ice cream

^1^ Monteiro, C.A.; Cannon, G.; Levy, R.B.; Moubarac, J.-C.; Jaime, P.; Martins, A.P.; Canella, D.; Louzada, M.L.; Parra, D.; with Ricardo, C., et al. NOVA. The star shines bright. World Nutrition 2016, 7, 28–38. ^2^ FFQ, food frequency questionnaire in PROtEuS.

**Table 2:** Selected characteristics of cases and controls participating in PROtEuS, Montreal, Canada (2005–2012).

Characteristics	Cases (*n* = 1919)		Controls (*n* = 1991)	
Age in years, mean (SD)	64	(7)	65	(7)
Ethnicity, *n* (%)				
French	1316	(69)	1154	(58)
Black	126	(7)	86	(4)
Asian	22	(1)	67	(3)
Other European	371	(19)	530	(27)
Greater Middle Eastern	42	(2)	100	(5)
Latino	33	(2)	31	(2)
Family income in $ CAD, *n* (%)				
<20,000	223	(12)	245	(12)
20,000–29,999	262	(14)	252	(13)
30,000–49,999	445	(23)	462	(23)
50,000–79,999	422	(22)	410	(21)
>80,000	425	(22)	428	(22)
Unknown	142	(7)	194	(10)
Education, *n* (%)				
Primary school of less	443	(23)	426	(21)
High school	572	(30)	578	(29)
College	313	(16)	375	(19)
University	588	(31)	610	(31)
Other	3	(0.2)	2	(0.1)
Body mass index 2 years ago (kg/m^2^), mean (SD)	26.8	(4)	27.1	(4)
Ever smoked, *n* (%)				
No	514	(27)	514	(26)
Yes	1404	(73)	1477	(74)
Overall physical activity, *n* (%)				
Not very active	431	(23)	488	(25)
Moderately active	522	(27)	558	(28)
Very active	965	(50)	945	(47)
Timing of last prostate screening test by PSA ^1^ or DRE ^2^, *n* (%)				
<2 years before index date	1903	(99)	1510	(76)
2–5 years before index date	1	(0.1)	154	(8)
>5 years before index date	0	(0)	81	(4)
Never screened	2	(0.1)	190	(10)
Unknown	13	(0.7)	56	(3)
First-degree relative with prostate cancer, *n* (%)				
No	1409	(73)	1736	(87)
Yes	447	(23)	199	(10)
Total calories/day 2 years before index date, mean				
(SD)	1989	(663)	1917	(646)
History of type 2 diabetes, *n* (%)				
No	1627	(85)	1593	(80)
Yes	289	(15)	395	(20)

^1^ PSA, prostatic specific antigen. ^2^ DRE, digital rectal exam.

**Table 3:** Adjusted ^1^ odds ratios (ORs) and 95% confidence intervals (CIs) for the association between food processing scores and prostate cancer risk in PROtEuS, Montreal, Canada (2005–2012).

Consumption Score by Category of Food Processing ^2^	1991 Controls *n*	All Prostate Cancers 1917 Cases ^5^ *n* Cases OR (95% CI)	Low-Grade Prostate Cancers ^3^ 1386 Cases *n* Cases OR (95% CI)	High-Grade Prostate Cancers ^4^ 530 Cases *n* Cases OR (95% CI)
Unprocessed/minimally processed foods				
Q1 (lower score)	498	527, 1.00 (reference)	376, 1.00 (reference)	150, 1.00 (reference)
Q2	497	509, 0.98 (0.81–1.19)	362, 0.97 (0.78–1.20)	146, 1.01 (0.77–1.32)
Q3	498	497, 0.95 (0.78–1.15)	366, 0.98 (0.79–1.21)	130, 0.88 (0.67–1.17)
Q4 (higher score)	498	386, 0.86 (0.70–1.07)	282, 0.90 (0.71–1.12)	104, 0.80 (0.59–1.08)
Processed foods				
Q1 (lower score)	498	527, 1.00 (reference)	376, 1.00 (reference)	150, 1.00 (reference)
Q2	497	486, 1.18 (0.96–1.45)	346, 1.18 (0.94–1.47)	140, 1.20 (0.90–1.61)
Q3	498	484, 1.09 (0.89–1.34)	361, 1.13 (0.90–1.41)	121, 1.13 (0.90–1.41)
Q4 (higher score)	498	533, 1.29 (1.05–1.59)	381, 1.27 (1.02–1.60)	151, 1.33 (0.99–1.78)
Ultra-processed foods				
Q1 (lower score)	498	527, 1.00 (reference)	376, 1.00 (reference)	150, 1.00 (reference)
Q2	497	495, 0.86 (0.66–1.11)	367, 0.85 (0.64–1.13)	128, 0.87 (0.59–1.27)
Q3	499	479, 0.95 (0.74–1.22)	342, 0.98 (0.75–1.29)	135, 0.87 (0.61–1.26)
Q4 (higher score)	497	516, 0.92 (0.72–1.17)	364, 0.89 (0.68–1.16)	151, 0.97 (0.68–1.38)

^1^ Adjusted for age, ethnicity, education, first-degree family history of prostate cancer and timing of last prostate screening. ^2^ Score taking into account the portions consumed by category of the NOVA classification presented by quartiles. ^3^ Prostate cancer cases with a Gleason score of 6 or lower or of 7 with a primary score of 3. ^4^ Prostate cancer cases with a Gleason score of 7 with a primary score of 4, or 8 or higher. ^5^ For one case, we had no information on Gleason’s primary and secondary patterns precluding classification into low- or high-grade cancer.
